# Philo-Medicus on Medical Degrees

**Published:** 1802-01

**Authors:** 


*To the Editors of the Medical and Phyfical Journal.*

GENTLEMEN,

IN two of the late Numbers of your valuable Journal, I troubled you with a few Remarks on the utility of publifhing an annual lift of Medical Practitioners. My defign has been happily happily accomplifhed; the Editors of the Britifh Almanack have been at confiderable pains in collecting and arranging this lift. But owing to a defect in their advertifements, and the late period at which they were publifhed, a very great number of names muft arrive too late for infertion. The lift will be more complete in the future editions.

In this matter, however, there is ftill an abufe. Some of the Univerfities, which have obtained Charters from Government for the purpofe of granting Medical Degrees, have fo degraded themfelves as to grant them to any perfon who can pay the common fees. A man with fifteen pounds in his pocket, and a certificate from two phyficians, however contemptible in their characters, is a Candidate fufficiently qualified to obtain a Degree! . . . . . . . . . . . . . .

In the Univerfities of Edinburgh and Glafgow, a ftudent cannot be admitted even to the Examinations without attending the whole of the medical claffes for a certain number of years. After giving fatisfaction on this head, he muft next undergo a very fevere examination. Befides, he muft publifh a Differtation on fome medical fubject, and defend the doctrines he has advanced before the whole Faculty. For an account of the different trials they muft go through, I refer your Readers to a very diftinct account of it in the laft volume of your Journal. (See No XXXII. p. 301.)

But at the Univerfities of St. A*******, and A*******, this is not the Cafe. Send the money and the *contemptible* certificate, and you have your degree by return of poft. I fay, *contemptible certificate*, for no man of character will be concerned, nor have his name feen in fuch difgraceful tranfactions. I have known the degree of Doctor of Medicine conferred on a man who had only ftudied one fhort feafon at the claffes! I have known it conferred upon men who, I believe, never ftudied at all! I have known men who bore the honourable title of Phyficians, proftitute their names in procuring fuch degrees for a fmall fum of money!

[ To be continued. ]

---

* Thefe terms, we believe, are not correct, and are certainly harfher than the caufe of truth can ever require. Ed.

State

